# Assessing the success of a research leadership programme for senior nurses and midwives: A mixed methods programme evaluation

**DOI:** 10.1002/nop2.2176

**Published:** 2024-07-17

**Authors:** Julie Christine Menzies, Rachel Ford, Catherine Henshall

**Affiliations:** ^1^ Paediatric Intensive Care Unit, Bristol Royal Hospital for Children University Hospitals Bristol and Weston NHS Foundation Trust Bristol UK; ^2^ Honorary Senior Research Fellow University of Birmingham Birmingham UK; ^3^ Visiting Fellow University West England Bristol UK; ^4^ Nursing and Midwifery Office, NIHR Leeds UK; ^5^ Oxford Institute of Nursing, Midwifery and Allied Health Research (OxINMAHR), Faculty of Health and Life Sciences Oxford Brookes University Oxford UK; ^6^ Research and Development, Warneford Hospital Oxford Health NHS Foundation Trust Oxford UK

**Keywords:** capability, capacity, clinical academic, mixed methods, programme evaluation, research midwife, research nurse, service evaluation, stakeholder interviews, survey

## Abstract

**Aims:**

In 2018 the National Institute of Health and Care Research, United Kingdom, launched a 3‐year Senior Nurse and Midwife Research Leader Programme to support nurse and midwifery research leaders to develop research capacity and capability within NHS organisations. We report the results of a service evaluation of the programme strengths, areas for improvement and achievement of programme aims.

**Design:**

Partially mixed, concurrent mixed methods programme evaluation, including: (a) meeting evaluation (survey), (b) annual evaluation (survey) and (c) qualitative stakeholder interviews.

**Methods:**

Survey results were quantitatively analysed using descriptive statistics. Interviews were audio‐recorded, transcribed, deductively coded using elements within the logic model and analysed using the seven‐stage framework analysis method.

**Results:**

Satisfaction with the programme was high (75%). The main perceived benefit of the programme was being part of a network. Challenges included accessing learning resources, lack of opportunity to network and lack of clarity about the programme aims. Meetings were evaluated as relevant and helpful (mean 93%), thought‐provoking (92%), inspiring (91%), at the appropriate level (91%) and aligned with the programme aims (90%). All meetings were ranked as highly beneficial by attendees (92%). Stakeholder feedback on the programme success reflected the importance of leadership, the programme design and content, ‘connection and community’ and communication with and about the cohort. Overall, the anticipated programme aims were met, evaluating well from both the perspective of those on the programme and the wider stakeholder group. There has been a lack of investment in schemes to support research leadership development for nurses/midwives. A novel programme to support nursing/midwifery research leadership was positively evaluated. The programme is a useful model to support future capacity and capability building for nurses/midwives. The work is reported with reference to the SQUIRE 2 and SRQR checklists. No patient or public contribution.

## 1 INTRODUCTION

Nurses and Midwives are the largest group of healthcare professionals and, as the main providers of patient facing care, are well positioned to improve service delivery and contribute to improved patient outcomes and satisfaction (Henshall et al., 2020). Despite growing recognition of the need for research to be the cornerstone of high‐quality, evidence‐based nursing practice (CNO, 2021), engaging nurses and midwives with research and implementing evidence‐based care has historically been challenging (Melnyk et al., 2010). This is partly due to a lack of protected time for nurses and midwives to undertake research training, as well as limited access to structured research career progression pathways (Avery et al., 2019; Trusson et al., 2019). This has reduced capability and capacity to progress as research leaders (Braidford & Terry, 2015). Other barriers to research career progression include a lack of funding, clinical academic leadership and effective partnership working (Henshall et al., 2021). Resultingly, nurses and midwives are often less embedded in the research agenda than their multidisciplinary team peers and have more limited research profiles. Limited opportunities for nurses and midwives to fulfil their research aspirations and contribute to local, national and international research priority setting processes, can have long‐term detrimental impacts on clinical care outcomes (Braidford & Terry, 2015). In addition, research leadership takes many different forms, encompassing research delivery and nurse‐led research, within academic, NHS and other settings. The level of support, recognition and profile given to nursing and midwifery research leadership roles is often understated (Carrick‐Sen et al., 2015), creating barriers for nurses and midwives who wish to pursue these roles.

## 2 BACKGROUND

To address some of these issues, and to enable nurses and midwives to directly inform, implement and deliver on healthcare priorities, in 2018 the National Institute of Health Research (NIHR) launched a 3‐year Senior Nurse and Midwife Research Leader Programme (SNMRL) known colloquially as the ‘70@70’ (Castro‐Sanchez et al., 2020; Henshall et al., 2020). The 70@70 SNMRL programme recruited 70 nurses and midwives in senior positions in English NHS Trusts (Agenda for Change banding seven and above), with the aim of realising their untapped potential to increase research capacity and capability, support the development of future research leaders and contribute to key NIHR priorities (Henshall et al., 2020). The NIHR programme objectives were:

- Make a significant contribution to the NIHR as a senior Nurse/Midwife research leader.
- Lead the development of NIHR‐funded staff and promote a vibrant research culture.
- Act as an ambassador for the NIHR.
- Exhibit research excellence.
- Contribute to growth (research activity/profile/engagement).
- Integrate Patient and Public Involvement and Engagement (PPIE) in research.
- Contribute to healthcare publications (lead/co‐authorship).
- Support the public health response to COVID‐19 (NIHR, 2021).

The SNMRL programme provided NMs with 2 days each week to focus on research priorities within their employing Trust, as a means of increasing nursing and midwifery research capacity, improving patient care outcomes and demonstrating research leadership. A central NIHR Nursing and Midwifery leadership team managed the programme, with oversight from a Steering group comprised of members of the leadership team, finance and web teams, programme facilitators and wider NIHR and Department of Health and Social Care (DHSC) representatives. Details of the programme development and implementation have been published previously (Henshall et al., 2020). SNMRLs were spread across England and were assigned to four regional hubs (Midlands, North England, London/South‐East and the South‐West), each led by experienced nurse/midwife clinical academic leaders or ‘facilitators’. A fifth hub provided mentorship and facilitation specifically for the mental health‐trained SNMRLs. The hubs met regularly, both online and face to face, throughout the programme to share and develop research initiatives and ideas, as well as to promote peer learning and discussion. Bespoke online resources and communications were provided through the NIHR Learn platform. National meetings were held with the entire cohort at least twice a year. The programme themes spanned from ‘Place’ in year 1, to ‘Power’ in year 2 and ‘Potential’ in Year 3 with material designed to support these, delivered by UK nursing and midwifery leaders and members of the Chief Nurse Officer team.

Following substantial investment in the programme, a comprehensive evaluation was required to identify any programme strengths that could be built on and replicated for future cohorts, as well as any areas for improvement.

## 3 THE STUDY

### 3.1 Aim

The aim of the programme evaluation was to explore SNMRL programme stakeholders' views on the programme to identify whether it was effective in addressing the programme development aims, as well as identifying any strengths and areas for improvement.

## 4 METHODS

### 4.1 Design

A pragmatic, mixed methods approach was adopted, founded on the principle that understanding the experiences of those engaged with or overseeing the SNMRL programme was important. Pragmatism strives to focus on what works best for understanding and solving problems (Brown & Dueñas, 2020) and on methods that work best in practice to answer specific questions. The plurality of qualitative and quantitative components was felt to be valuable to provide a deeper insight (Kelly et al., 2018). The project therefore adopted a partially mixed, concurrent, equal status design, with quantitative and qualitative parts conducted concurrently, with equal weighting, and without being mixed or compared until both sets of data had been collected and analysed. The project was classified as a service evaluation, evaluating how well the programme was achieving its intended aims (Twycross & Shorten, 2014). It was undertaken with the purpose of evaluating the programme through a mixed methods approach, to inform decision‐making about the current and future programmes. In line with service evaluation standards, the work is reported with reference to the Standards for Quality Improvement Reporting Excellence (SQUIRE 2) checklist (Ogrinc et al., 2016). We also reference the Standards for Reporting Qualitative Research (SRQR) (O'Brien et al., 2014) for the qualitative interviews.

### 4.2 Theoretical framework

**FIGURE 1:**
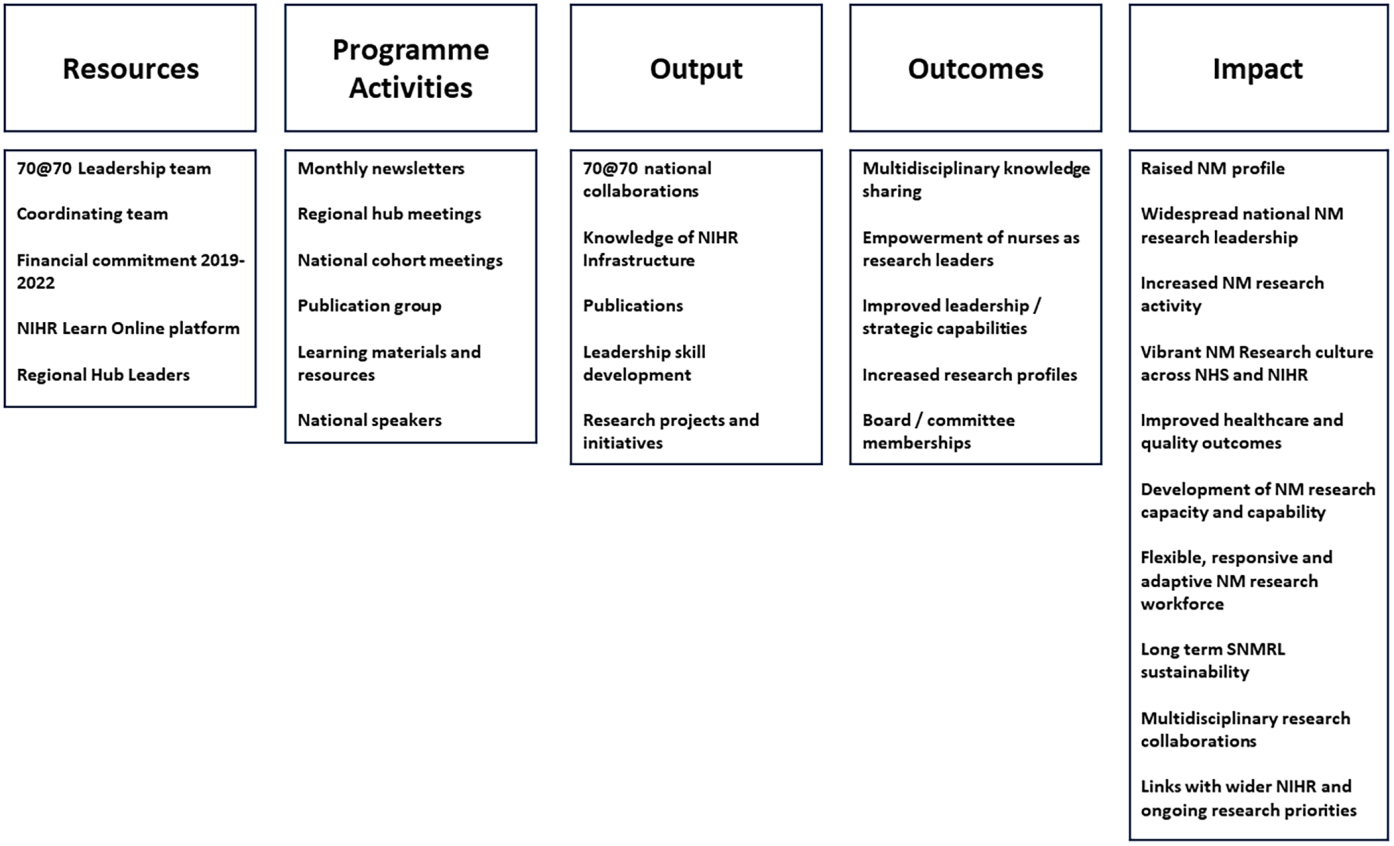
Logic model to show inputs and projected outputs, outcomes and impact of the SNMRL Programme.

Logic modelling was employed as a theoretical framework to underpin the evaluation. Logic models provide stakeholders with clear processes or sequences of related events that connect building blocks of a planned programme with its desired outcomes and impact (Kellogg Foundation, 2004) (Figure 1). The model provided a useful way to identify the study resources, programme activities, intended outputs, outcomes and anticipated impact. This supported the development of the evaluation methods; from identifying the stakeholders, to the aspects of the programme to be evaluated, to the development of appropriate tools to use within the evaluation. There was also recognition of the importance of a mixed methods approach, incorporating both quantitative and qualitative approaches.

### 4.3 Sampling and recruitment

The evaluation was conducted to explore the experience of SNMRLs appointed to the programme and working within English NHS Trusts, as well as the perspectives of other key stakeholders.

### 4.4 Population and sample

There were 70 SNMRL's appointed to the scheme in 2019. At the end of year one there were 66 nurses and midwives remaining, 63 at the end of year two and 60 at the end of year three. For the qualitative programme review (interviews) conducted at the end of year two we aimed to recruit 20–30 participants', 15 SNMRLs and 15 wider stakeholders (Chief Nurses, NIHR team and steering group).

### 4.5 Inclusion and exclusion criteria

All SNMRLs on the programme were eligible to complete the annual survey evaluating the programme. All SNMRLs who attended national training/meetings during years two and three (meetings 6–11) were eligible to complete a meeting evaluation. Meetings 1—5, conducted May 2019–March 2020 were not formally evaluated. Participants for the qualitative interviews were from three key stakeholder groups:

- individuals appointed to the scheme (SNMRL) (*n* = 66),
- individuals invested in the outcomes of the programme at local level (Chief Nurses within NHS Trusts)
- individuals with responsibility for programme delivery and development (NIHR team and SNMRL steering group)

To ensure a representative and diverse spread of SNMRLs amongst participants, a purposive sampling framework was employed to recruit from the 63 SNMRL on the programme in year two. A proportion of nurse and midwives from different NHS pay scales (bands 7–8 d), genders, ethnicities, professional backgrounds and job titles and different hubs were invited. SNMRL were purposively sampled to ensure there was representation from acute, community and mental health settings and different sized NHS Trusts. The trust size was calculated using data available in the public domain on the number of staff per Trust (NHS Digital, 2019). Using this data, SNMRLs from small, medium and large trusts (*n* = 25) were invited to participate.

Participants' representing the wider stakeholder groups (total *n* = 24) were invited through a variety of means. The sampling frame for Chief Nurses was those who had responded to the year one survey evaluating the impact of SNMRLs within their organisations (*n* = 14, 26% response rate). Individuals were purposively sampled to ensure representation from Chief Nurses working within small, medium and large organisations, and representing the four geographical locations defined by the regional hubs (*n* = 6). Other invited participants included the NIHR team (*n* = 4), programme coordinators (*n* = 2), hub facilitators (*n* = 5) and Steering Group representatives (excluding those in roles already listed) (*n* = 7).

### 4.6 Data sources/collection

There were three key programme evaluation components.

#### 4.6.1 Programme design survey (annual)

A brief survey was designed by the Nursing and Midwifery Programme Director (Appendix S1). The survey was developed from feedback gained during year one meetings and through consultation with the steering group. It was piloted with members of the leadership team. Two questions asked respondents to rank on a scale of 0–100 (0 being the lowest and 100 being the highest) their: (a) satisfaction with the programme and (b) their understanding of their role on the programme. The year one results have previously been published (Henshall et al., 2020). The survey was revised for years two and three with respondents offered pre‐populated options derived from the year one survey, categorised as challenges and benefits to engaging with the programme which enabled comparison across the 3 years. The survey was distributed to all SNMRL at the end of years one (*n* = 66), two (*n* = 63) and three (*n* = 60). The survey was anonymous, so it was not possible to compare individual respondents' views across the 3‐year period.

#### 4.6.2 Meeting evaluation survey

The meetings were scheduled as half day events, with a variety of speakers and programme content related to nursing and midwifery leadership. The survey purpose was to collect data on the perceived value of the meetings. This included five questions asking participants to rate the meeting relevance, ability to inspire, the pitch of material, stimulation of reflection and alignment to the programme aims (Appendix S2). The survey was distributed to all SNMRL who attended the meetings during year two (*n* = 63) and year three (*n* = 60) within 1 week of the meeting, with a reminder at 2 weeks.

#### 4.6.3 Stakeholder interviews

Stakeholders were invited to participate through email invitation (with participant information sheet) sent by the evaluation team, with a reminder sent after 2 weeks. If stakeholders agreed to take part, an interview date was arranged, and a consent form was signed before their interview took place. A topic guide (Appendix S3) developed from the logic model (Figure 1) was used to structure and inform the interviews and included questions such as: what do you think have been some of the challenges and barriers to successfully setting up and delivering the programme? What features of the programme did you like the best?

Semi‐structured interviews were conducted by the evaluation lead between June 2021 and October 2021. These lasted between 30 and 75 min, were conducted by video‐call and recorded. Participants were allocated a unique study identifier and recordings were saved to a secure NIHR drive with restricted access to the research team. Interviews were transcribed, anonymised, and imported into NVivo12 for analysis.

### 4.7 Analysis

Programme design survey responses were exported and analysed quantitatively in Microsoft Excel. Where participants ranked their satisfaction and understanding of their role on a scale of 0–100, a mean percentage was calculated allowing comparison over the 3 years. Free text responses were imported into NVivo and categorised to ‘challenges’ and ‘benefits’ of the programme and used to provide additional information about participants responses. Meeting evaluation responses were analysed in Microsoft excel. Participant responses agree and strongly agree were combined for analysis and reported as the number of people and percentage who agreed. Agreement of participants about the value of each session on the five elements under review was calculated (mean percentage) and a mean score of each meeting was also calculated.

Stakeholder interviews were thematically analysed using the framework approach (Gale et al., 2013). This was important to compare the perspectives of the three participant groups. A deductive approach to coding was used, with elements listed within the logic model ‘resources’ and ‘programme activities’ used as coding categories and additional codes added to reflect programme challenges and strengths. After coding the first few transcripts the resulting codes were reviewed and refined and incorporated into a working analytical framework by two members of the research team. This was then used to code the remaining transcripts. Data were charted into the framework matrix, facilitating movement between the data and the emergence of the themes. The process ensured links between the original data and findings were transparently maintained, enhancing rigour (Smith & Frith, 2011).

### 4.8 Ethical considerations

In accordance with the NHS Health Research Authority decision tool, the work was not defined as research (NHS HRA, 2021); therefore, ethical approvals were not required. The work was classified as a service evaluation, evaluating if the programme successfully achieved the NIHR aims. The protocol for the conduct of this evaluation was reviewed and approved by the NIHR Steering Group in January 2021. Anonymity of participants was maintained as transcripts were de‐identified and any details regarding role or location were removed before sharing.

### 4.9 Rigour

To enhance the trustworthiness the evaluation lead was appointed specifically to undertake the interviews and complete analysis of the quantitative and qualitative data. They were not known to any of the cohort or stakeholder group and had no investment in whether the programme achieved the aims. This was counterbalanced by the other team members who were familiar with the SNMRL programme and provided a means of clarification about the programme and supported reflections. The evaluation lead maintained detailed notes to support preliminary analysis and the dependability of results. Transcripts were not returned to members; however, a summary of themes were shared with participants' and the wider SNMRL and stakeholder group for confirmation and participants' words were used within the final report to provide rich description.

## 5 FINDINGS

### 5.1 Programme design survey

The survey response rate was 39 (59%) in year one, 48 (76%) in year two and 33 (55%) at the end of year three. The SNMRL cohort ranked programme satisfaction highly, with a mean score across all 3 years of 75%. Free text comments from all 3 years (denoted as A‐yr 1, B‐yr 2, C‐yr 3) reflected on the programme opportunities:

Excellent programme providing leadership skills, knowledge, and valuable networking opportunities (SNMRL 33‐B)

**FIGURE 2:**
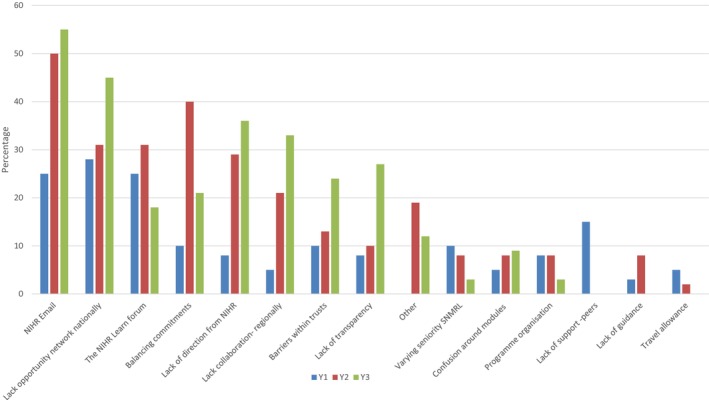
Challenges selected by the SNMRL cohort and reported for years one–three of the programme.

The highest reported challenges over the course of the programme are shown in Figure 2. These reflected issues associated with the requirement to use NIHR email addresses, and therefore, managing multiple email accounts (*n* = 52), as well as problems accessing the NIHR Learn online forum (*n* = 31):

The course was excellent but online resources were hard to access and stopped after year one which was a shame (SNMRL 10‐C)

COVID‐19 had a significant impact on the final 2 years of the programme, with staff redeployment and role changes and all meetings were converted to virtual meetings. This was reflected in the wide reporting of challenges across all 3 years about the reduced networking and collaboration opportunities – ‘*Not enough opportunities to network as a national group*’ (*n* = 41) and *‘not enough collaboration between regional hubs*’ (*n* = 23). Free text comments reflected these:

The pandemic limited national networking opportunities (SNMRL 13‐C)

Great opportunity and commitment from hub leads, but frustrating that face to face input was hampered by COVID (SNMRL 5‐C)

Lack of direction/clarity from the NIHR was an issue reported throughout the 3 years (*n* = 29). For some this related to the lack of aims and objectives at the start of the programme:

The direction at the start was not clear, I expected more of a steer (SNMRL 41‐A)

For others it was more about mixed messages about the programme purpose and objectives:

At times there has been confusion over the vision for the programme (SNMRL 4‐B)

Issues around communication within and about the programme were commented on. For some SNMRLs, they felt there was insufficient information available about what other SNMRL were working on or developing:

Not enough communication on what others doing. Felt out of the loop at times (SNMRL 10‐C)

Others were frustrated about a lack of communication about the programme to an external audience and the lack of visibility of the scheme:

Lack of website is a frustration. It would have helped communication and reach outside of 70@70 (SNMRL 34‐B)

The main perceived benefits of undertaking the programme were also reported by participants' (see Table 1). The most reported benefit was being part of a network:

Well‐structured programme that has provided opportunities to develop a number of links and networks (SNMRL 6‐B)

**TABLE 1:** Summary of the beneficial aspects of the programme.

Beneficial aspects of the programme (*n*, %)	Y1 (*n* = 39, %)	Y2 (*n* = 48, %)	Y3 (*n* = 33, %)
Being part of a network	39 (100)	44 (92)	31 (94)
Ring‐fenced time	14 (36)	34 (71)	26 (79)
Control over own time and objectives	8 (21)	26 (54)	23 (70)
Communication from NMO	5 (13)	22 (46)	13 (39)
Resources on NIHR learn	7 (18)	4 (8)	1 (3)

The value of networking grew in importance over the duration of the programme; from 74% at the end of year one to 94% by the end of year three. Having protected time was the next most positively viewed programme benefit. This became more valued over the course of the programme, increasing from 36% in year one to 79% by the end of year three:

Fantastic opportunity for protected time to develop new initiatives (SNMRL 13‐B)

Having control over time and objectives became increasingly important to participants, increasing from 21% to 70% by the end of year three. SNMRL's valued freedom within the programme to focus on local needs and priorities:

Flexibility to develop initiatives appropriate to your organisation (SNMRL 40‐C)

The benefits of communication from the NIHR leadership team were recognised (n = 40), with comments noted about the benefit of communication and leadership from the Programme Director and Hub facilitators:

Clear guidance and support from hub lead (SNMRL 18‐A)

### 5.2 Programme meetings evaluation

Formal evaluation of the meetings took place from year two onwards. Two meetings were cancelled due to the pandemic therefore responses from attendees at five meetings were therefore eligible for evaluation (Table 2).

**TABLE 2:** summary of meeting evaluations (year two and three).

Years 2 and 3	Session 6 (*n* = 40, %)	Session 7 (*n* = 31, %)	Session 8 (*n* = 50, %)	Session 10 (*n* = 45, %)	Session 11 (*n* = 46, %)	Mean score (%)
The meeting was relevant and helpful to me as a research leader	39 (98)	22 (71)	49 (98)	45 (100)	45 (98)	93
The speakers were inspiring and provided me with useful insights	37 (92)	22 (71)	47 (94)	45 (100)	46 (100)	91
The content and topics were pitched at the right level	37 (92)	21 (67)	47 (94)	45 (100)	47 (100)	91
The session was thought‐provoking and make me reflect on my own role and purpose as a research leader	38 (95)	23 (74)	48 (96)	44 (98)	45 (98)	92
The session aligned well with the overall aims and scope of the programme	35 (88)	22 (71)	47 (94)	45 (100)	45 (98)	90
Mean score per hub meeting	93	71	95	100	99	

A mean of 42 responses from SNMRL were received per meeting. Respondents agreed that meetings were relevant and helpful (mean score 93% across all five meetings), thought‐provoking (92%), inspiring (91%), at the appropriate level (91%) and aligned well with programme aims (90%). When each meeting was reviewed attendees had high agreement that the meetings had satisfied all five aspects, with a mean score of 92% agreement. One meeting scored lower than the others with a mean score of 71%. This meeting took place in early 2022 as the UK addressed further COVID waves and had the lowest attendance rate (*n* = 31).

### 5.3 Stakeholder interviews

#### 5.3.1 Participants

In total, 34 of 46 invited participants were interviewed. These included 17 of 21 invited SNMRLs and 17 of 25 invited stakeholders (see Table 3 for a summary of invited participants and Table 4 for a summary of the SNMRL participants).

**TABLE 3:** Summary of all invited interview participants.

Participant and unique identifiers (SG 1–17)	Role description	*N* (% of sample invited)
NMO leadership team (SG1‐4) (*n* = 4)	Responsible for the set‐up, implementation and delivery of the programme	4 (100)
Programme delivery support team (SG5‐6) (*n* = 2)	Included: administrative and logistical support, finance and website development and resources	2 (100)
Steering group committee members (SG7‐9) (*n* = 13, 7 invited)	Steering group members who provided feedback and direction on all elements of the programme	3 (43)
Chief nurses (SG10‐12) (*n* = 14; 5 invited)	Chief Nurses within organisations with at least one SNMRL	3 (60)
Facilitators (SG13‐17) (*n* = 5)	Geographical or speciality ‘hub’ leaders	5 (100)
SNMRL cohort (*n* = 63, 21 invited) (SNMRL 1–17)	Purposive sample from SNMRL cohort in year two	17 (81)

**TABLE 4:** characteristics of the SNMRL cohort and the interview participants.

Demographic		SNMRL cohort year 2 (*n* = 63)	SNMRL participants (*n* = 17)	% of cohort sample
Banding	7	8	4	50
	8a	19	5	26
	8b	14	2	14
	8c	8	2	25
	8d	2	2	100
Gender	Male	4	2	50
	Female	59	13	22
Profession	Nurse	57	15	26
	Midwife	6	2	33
Area of practice	Acute	50	11	22
	Community	7	2	29
	Mental Health	6	2	33
Ethnicity	BAME	7	4	57
	Non‐BAME	56	13	23
Size of trust	Small	31	9	29
	Medium	17	4	24
	Large	14	2	14

**FIGURE 3:**
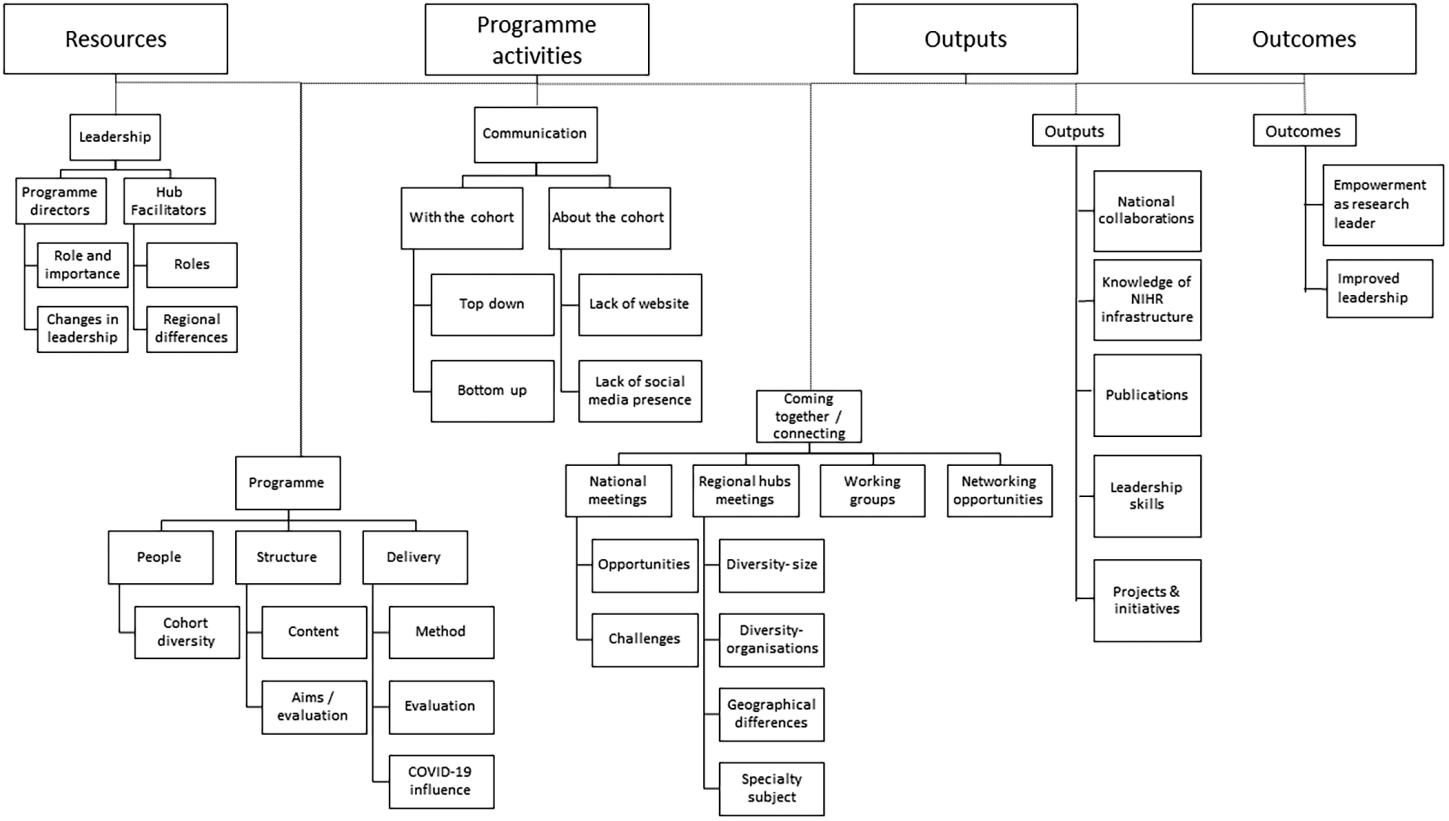
Coding tree.

Coding and their links to the key elements within the logic model are outlined in Figure 3. From this coding process four key themes were generated.

**FIGURE 4:**
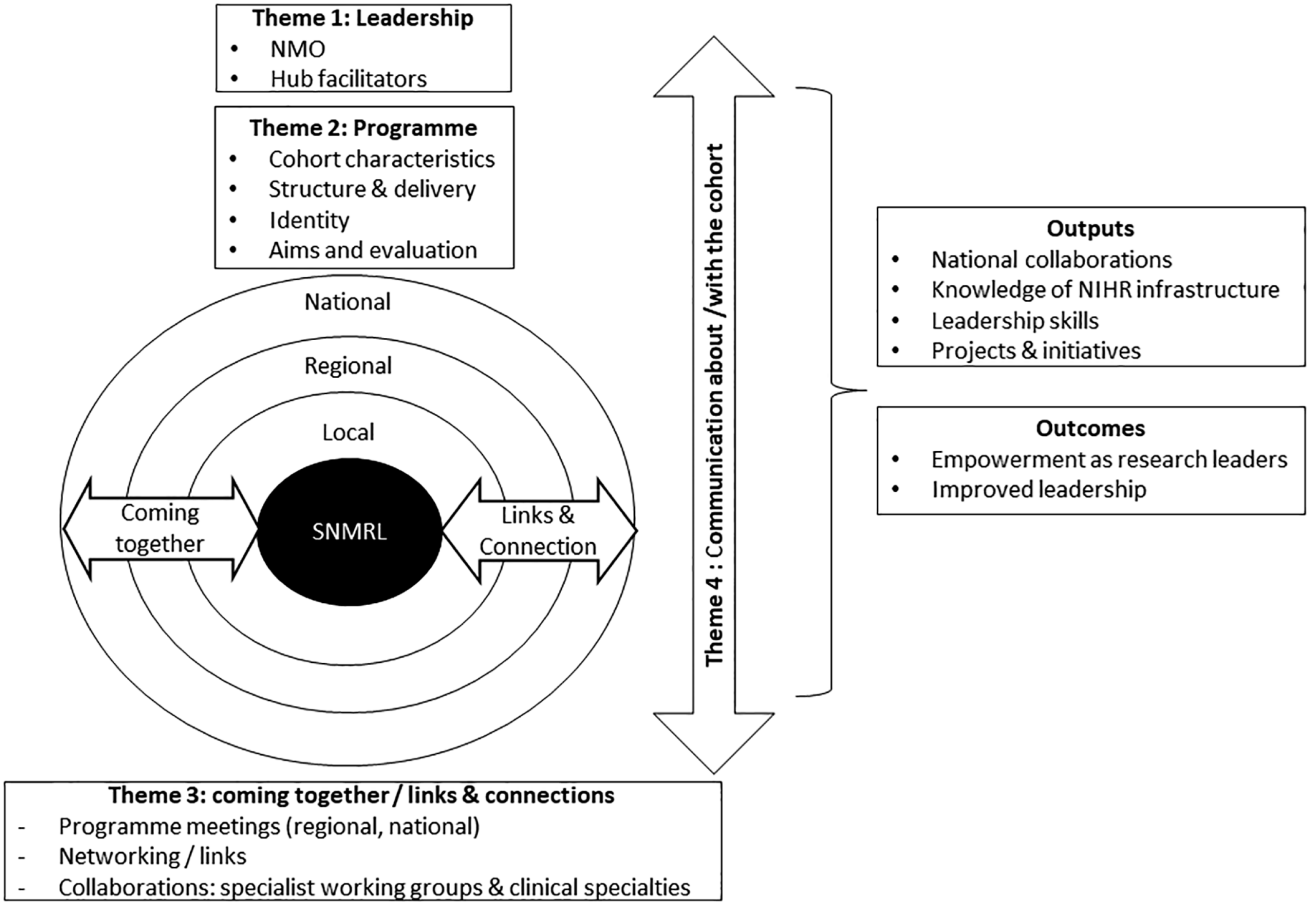
Summary of the four themes summarised from the qualitative interviews with stakeholders.

The four themes were summarised as ‘the role of leadership’, ‘programme identity and delivery’, depicted at the top of Figure 4 as the two elements provided by the NIHR. The appointed SNMRL are depicted at the centre, with rings around them to represent local, regional and national levels, with arrows indicating the networking or ‘coming together’ and ‘links and connections’ formed (theme three). ‘Communication’ was a cross‐cutting theme, reflecting communication *with* the cohort from the leadership team and externally *about* the cohort. Outputs and outcomes links to the anticipated outcomes envisaged in the logic model and demonstrates achievement of key elements. Findings from each theme are described in more detail.

##### Theme 1: The role of leadership

This theme related to the importance of leadership within the programme, both from the programme leadership team and the programme facilitators.

###### The leadership team

Twenty‐eight interview participants (including representatives from all the participating stakeholder groups) spoke of the importance of the leadership team.

I've found our [programme director] to be absolutely super – very supportive all the way [SNMRL2]

The biggest challenge to leadership reflected the part‐time nature of the role.

Each of the leaders have been excellent and committed to the programme. But none of them have had enough time because the role is only funded part‐time… they've done a good job, without much support [SG 17]

###### Changes to leadership

Over the course of the programme there were three programme directors and participants expressed differing views towards these changes. Some participants felt that the changes in leadership had positively impacted on the programme, with each of the leaders bringing different skill sets and experiences:

I think it has been incredibly good to have those groups of people. Different people leading have brought different priorities and different strengths [SG 14]

However, there were reflections that the changes in leadership had impacted on the aims and direction and continuity of the programme:

It did feel like every time there was a change, there was a slight emphasis in terms of the programme expectations or how things were being recorded…I think, had we had one person oversee it from start to finish, we would have had a greater consistency around that [SNMRL 7]

###### The role of the facilitators

The facilitators were considered by many to be a key programme strength. Benefits of the facilitator role included providing a means to feedback to the leadership team, input in the initial programme development, the provision of mentorship and access to knowledge, experience and expertise:

Our facilitator was grand. She was very, very knowledgeable and she'd basically done what we are striving to do at her own hospital without the resources we currently have, so that was good to have her as a point of reference [SNMRL 12]

While there were changes to the central leadership team throughout the programme, the facilitators remained constant throughout the programme duration which was felt to have been important:

They've provided a level of consistency across the programme which has not been present in the senior leadership team [SG14]

##### Theme 2: Programme identity and delivery

The programme content was a central part of discussions and was therefore reflected in a theme – ‘programme identify and delivery’. This included reflections on who it was for, how it was structured, the programme aims and how impact was measured.

###### Recruitment and diversity

Participants reflected on the fact that SNMRLs came from a variety of different organisations, backgrounds, bands, and levels of seniority and many felt this helped with a cross‐pollination of ideas and perspectives. This diversity was felt by many to be a positive aspect of the programme:

For those that came in more academic [roles], to introduce them to the operational challenges of clinical research, and clinical research nursing and midwives and vice versa, so that there would be that synthesis of ideas [SG 1]

However, some participants reported that the diversity of the SNMRLs in terms of roles, seniority and banding posed a challenge to the programme, particularly from those in research delivery roles as many felt the programme was geared more towards clinical academics:

There's such a big discrepancy in the cohort itself, there are some…publishing continuously, doing research. And for me, whose more into the delivery and getting the word out? I feel like I'm just at the bottom of it [SNMRL 8]

The diversity of the cohort was reported to be challenging at times from a leadership perspective:

…the cohort are really diverse and interesting on many levels, but it also has been challenging in terms of thinking about how to pitch things at the right level because what might suit somebody…may not suit somebody else who's at a higher or lower level or a different stage of their career [SG 4]

A further source of diversity was the research infrastructure within SNMRLs' organisations. Some SNMRLs came from organisations with well‐developed research infrastructure, whereas others were using the programme as an opportunity to develop a new research infrastructure:

Those that had less experience or less infrastructure struggled more at the beginning, but at times felt that they were achieving less than others because they were comparing themselves to others in different roles, different experiences with different organisational infrastructures [SG 2]

###### Programme delivery

In line with feedback from the programme evaluation survey, 20 interview participants commented on the learning resources and the NIHR Learn platform and the challenges surrounding emails. Some commented that the material within the NIHR Learn platform was of a high quality:

The content when you actually get there is very helpful and interesting and valuable [SG 14]

However, most participants felt that the NIHR Learn platform was difficult to access and navigate, particularly from NHS computers. For at least one SNMRL, this meant that they could only access the platform on their personal computer at home. Many respondents noted that they already had multiple email addresses, and so having to access another email address purely for the purposes of the programme posed a further barrier:

It's on a totally different platform, NIHR, you had to use NIHR emails. I currently have about four email addresses as it is [SNMRL 12]

Due to these issues, in year two, the requirement to use the NIHR email and Learn platform was removed and resources were distributed via SNMRL's preferred email addresses.

###### Programme structure and evaluation

To cater to the diverse range of programme participants, the programme had to be flexible with the aims and objectives. For many this flexibility was important:

I guess because we were all coming from different perspectives, it would have been impossible to say that these are the core aims and objectives of what you will need to achieve… The fact that you were able…to set your own aims and objectives, really was key [SNMRL 14]

However, there was recognition that this caused confusion as to what the programme was, what it was aiming to achieve and how to measure impact:

It wasn't clear to me right at the beginning when the programme was designed. What I always asked at the time was ‘What are these people going to do?’ And I think that has been one of its challenges [SG 8]

##### Theme 3: Coming together and connecting

A key theme from participants reflected the importance of the programme in creating an opportunity for nurses and midwives s to network and connect with each other.

###### National meetings

Twenty‐three interview participants commented on the SNMRL national meetings and speakers. National meetings were viewed as opportunities for SNMRLs to unify and interact around a common theme or purpose and were reflected on positively:

It gives you that sort of inspiration, you know? You hear from very expert speakers; you get tips from people who are sort of delivering high‐level leadership courses [SNMRL10]

###### Regional hubs and meetings

Twenty‐seven interview participants described the value of the hub meetings. Most SNMRLs felt that they provided opportunities for the cohort to come together, provide peer support for each other, discuss local/regional issues, learn more about work that was taking place locally and to think about how ideas could be implemented in their own organisation:

We get so much from our regional networking and catching up and getting ideas… listening to how others have done stuff… to get things done in their own organisations [SNMRL13]

One of the facilitators commented that the regional hub meetings had been effective in forming relationships between disparate groups that would never have collaborated were it not for the programme:

In regions, there are natural synergies and unnatural ones…by bringing them together we've actually created something that I think is quite valuable [SG15]

Although defining the hubs by geography was viewed favourably by many of the participants, there were reports that some hubs were too geographically spread out:

Our hub has got geographical issues…there's a few up in [region] and then there's [another region], so they work together. And then there's me on my own. I mean, I've got over it, but I think it would have been nicer to maybe have some more people to work with [SNMRL16]

One hub focused specifically on mental health nursing and this specialty forum was perceived to be particularly effective and beneficial for members:

I think that's been the most beneficial for me, because mental health feels like it's in a different place to…general nursing or midwifery, so I think that's been the key [SNMRL 7]

Other specialty specific connections had developed organically within the programme, but more formal implementation was felt to be required for future programmes. This view was shared by one of the midwives on the programme:

It took us a while to find our feet…I think if there had been some dedicated facilitation by somebody from the same professional background, we might have got to that a bit sooner [SNMRL17]

###### Working groups

Several participants discussed the formation in year two of 13 separate working groups, designed to review specific aspects around nursing and midwifery research capacity and capability. The working groups were reported to have facilitated greater national connections and offered opportunities to connect with other SNMRLs with complementary research interests:

What it has offered is, people who may be beetling away in individual organisations, a network…It has got people [working] beyond their organisations [SG15]

Although the working groups were overall felt to have been beneficial, there were several comments about a lack of clarity and communication around their set‐up. One SNMRL felt the groups were poorly publicised and were unaware of their existence until it was too late to become involved in a meaningful way:

I only found out accidentally by one of the people in my hub that there was a few of these different workstreams going on…I wasn't part of any of them…I think that would [have] been better, if the hub leaders and the national [leaders] could have communicated that a bit better [SNMRL16]

###### Networking opportunities

The programme was reported by many participants, to have provided an excellent opportunity for networking and collaboration that extended beyond the hubs meetings and had enabled SNMRLs to engage and identify shared interests or specialist areas of practice. This had led to collaborations beyond the working groups:

There's no way that without the programme all of the connections and networks and friendships, but also working relationships and projects and all of those things would ever have been made if it wasn't for the programme [SG4]

###### Cross‐cutting theme four: Communicating with and about the cohort

Nineteen participants commented on communication between SNMRLs and the leadership team and generally there was satisfaction with the communication from the leadership team:

The participants know that if there's anything they want circulating they can ask to put it in that newsletter. I think they've got a lot out of it as well… they've been able to share their work across the cohort, and yeah, it's worked quite well [SG5]

Communication about the work of the cohort was also regarded as important but was regarded as problematic by many participants. The lack of SNMRL profile was viewed as a fundamental weakness with the programme and was raised repeatedly across all stakeholder groups. No online platform existed through which SNMRLs could promote and share their research initiatives, projects, and achievements:

I think there's a large issue with the website and the way this is communicated outside of the group… There's no representation on the NIHR website itself. No signposting to the 70@70 group, there's no national directory of 70@70 members… and what our responsibilities are, I… think that's a big failing [SNMRL 1]

## 6 DISCUSSION

The aim of the evaluation was to explore stakeholders' views on the NIHR SNMRL programme, review whether it was effective in addressing the programme development aims, as well as identifying any strengths and areas for improvement. The results suggest that overall, the SNMRL programme was perceived favourably by stakeholders. The interview findings triangulated well with the annual programme design survey and meeting evaluation results. Strong satisfaction scores for every meeting (mean score 92%) were also reflected in high satisfaction with the programme overall (mean score of 75%) and this was also reinforced in the interview findings. Issues relating to the challenges of accessing learning materials, initial programme clarity, the loss of face‐to‐face networking with other SNMRLs were evident across the programme design and interviews with stakeholders. There was also concordance about the benefits of the programme and the programme delivery. The programme design survey highlighted how much the opportunity to network and collaborate regionally and nationally and the leadership of the programme was valued and the meeting evaluations demonstrated that SNMRL felt the programme material and resources were relevant, helpful, inspiring, thought‐provoking and supporting their growth as research leaders. The interviews confirmed these benefits and offered more insights, expanding on the value of the leadership team to highlight the key role of the regional programme facilitators. The interviews also identified the importance of the programme structure and content, but also highlighted the influence of the people recruited to the programme and the benefits and challenges associated with the diversity of the SNMRL cohort. The importance of clear aims communicated from the outset was also referred to by interview participants and there was also reference to the fact that without this evaluation of SNMRL achievement was difficult. Challenges or areas for improvement and benefits or successful elements within the programme could be summarised by the four themes identified within the interviews: leadership, programme, coming together and connection and communication.

### 6.1 Leadership

The direction and leadership provided by the leadership team and the hub facilitators was a consistent feature. Leadership is vital in modern healthcare settings for improving the quality of healthcare provision and productivity (Kumar & Khiljee, 2016). Good leaders can create conditions that enable change and innovation to occur across the healthcare system (Edmonstone, 2013). Quality of care and organisational performance are directly affected by the quality of leadership and the improvement cultures that they create (Care Quality Commission, 2017). Within the UK the NHS Long Term Plan (2019) recognises the importance of nurturing the next generation of leaders through coaching and mentorship. Coaching and mentorship have been shown to produce improvements in care quality, evidence‐based decision‐making, leadership skill development, accountability, and staff satisfaction (Manzi et al., 2017). However, this is challenged by a lack of highly skilled senior leaders within the NHS (Anandaciva et al., 2018). For many SNMRLs, the leadership from the NMO and the facilitators was the only access they had to appropriate mentorship and/or coaching. Access to high profile, approachable research leaders who can provide role modelling was seen as a key strength of the programme. Access to role models can provide reassurance through their own prior experiences and stimulate growth, reflection, and learning (Felstead & Springett, 2016). This is key to ensuring the pipeline of research leaders is nurtured and developed across the international nursing and midwifery community. The programme has applicability and relevance to an international audience. Increasing nursing and midwifery research capacity and capability, enhancing the standing of the professions, and strengthening the voice of nurses and midwives to question, lead and implement changes are universal aspirations (Carrick‐Sen et al., 2019; Paterson & Strickland, 2023). Healthcare systems in many countries can learn from the strategic approach of the NIHR SNMRL programme, by considering the best ways to provide nurses and midwives with the tools, resources, and confidence to step into the research arena and actively contribute to research policy and practice (Henshall et al., 2020).

### 6.2 The SNMRL programme

SNMRLs included both clinical academics and those in research delivery roles, with a small number spanning both. This diversity proved to be positive from the perspective of driving both agendas – supporting capacity building across both areas and stimulating discussion and understanding of their reciprocal value. With the number of patients recruited into interventional studies identified as the variable most associated with improved Care Quality Commission rating and reduced mortality rates (Jonker & Fisher, 2018), research is recognised as a vital component of high‐quality services (Harding et al., 2016). As a result, there is growing recognition of the importance of expanding the research delivery workforce and improving understanding about the specialty of clinical research nursing and career opportunities for research nurses and midwives (Jones et al., 2021; Kunhunny & Salmon, 2017).

There have also been challenges to stimulating clinical academic growth. Despite a national drive to increase the number of nurses, midwives, and allied health professionals in clinical academic positions by 2030 (Carrick‐Sen et al., 2016), this situation has been slow to develop and there has been a lack of sustained and cohesive implementation of clinical academic research pathways (Henshall et al., 2021; Pattison et al., 2021). A situation also recognised within the international literature (Paterson & Strickland, 2023). Our findings suggest that the SNMRL programme was successful in promoting mutual respect and understanding of the range of ways nurses and midwives can contribute to clinical academic and research delivery opportunities within their organisations. This promotion aligns to several key policy initiatives, including from the NIHR (NIHR, 2021) and NHS England's Chief Nursing Office strategy (CNO, 2021). The CNO strategy outlines a clear plan to create a people‐centred research environment where nurses are empowered to lead, participate in, and deliver research. Key elements within this are to release nurses research potential, build the best research systems and develop nurse research leaders of the future. The SNMRL cohort working across England are now well placed to help operationalise and translate this strategy into action.

The diversity of the SNMRL cohort was also recognised as one of the programmes challenges, particularly tailoring the programme and providing support to a group with such a wide range of learning needs. Effective leadership of diverse teams requires proactive attention to team needs and adequate management of inter‐group processes (Homan et al., 2020). Although this can be challenging, leaders who recognise and proactively address team diversity and needs reap the benefits. Future SNMRL programmes could benefit from considering these requirements at the outset to ensure that the range and breadth and depth of skills possessed by different SNMRLs are harnessed and enabled, rather than being viewed as challenging and difficult to coordinate and lead. The findings also indicated the importance of clearly established and consistently applied aims and objectives from the outset of the programme. Goal setting is fundamental to organisational management yet is often not done well (Ogbeiwi, 2021). Future programmes should strive to establish clear aims and objectives which link to key performance indicators, with clear communication from the outset of the programme. This would not only help to support goal attainment but will also minimise the impact of any changes to programme leadership.

### 6.3 Connection and community

Networking opportunities were widely recognised as one of the greatest benefits of the programme. Networking is defined as building, maintaining and using relationships to enhance career success (Wolff & Moser, 2009). Networking has been identified as the most robust predictor of career success (Blickle et al., 2009) and for those with an academic component to their job, networking is becoming increasingly important to provide advice and support and connections within their clinical specialty (Ansmann et al., 2014). The SNMRL programme provided a means for SNMRL to network at a number of levels; as individuals with specific interests, similar roles or working within similar specialities, within their organisation – making new connections within their organisation to address programme aims and regional and national level connections on work of regional and national importance. These connections helped active‐bridge building; overcoming divides such as profession, organisational role and banding which limited intra and inter organisation connections. This is vital in creating larger, more resilient professional networks (Cunningham et al., 2012). Coming together was challenged by COVID‐19 and the cessation of all face‐to‐face meetings which impeded networking locally, regionally and nationally. Although there is evidence about the benefits of virtual conferences (Rubinger et al., 2020) this is not necessarily applicable to the SNMRLs. The SNMRL programme was developed in a pre‐COVID‐19 pandemic world, where face to face networking and communication was the default setting. As such, the programme was set up with geographical hubs that would allow for this. Now that virtual and remote working are more widespread, this would facilitate a move to hubs based on specialty as opposed to geography for any future programmes. The mental health hub evaluated overwhelmingly favourably, and this could provide a useful model for future programmes. However, opportunities for face‐to‐face contact should be provided where possible to counterbalance some of the challenges with remote meetings.

### 6.4 Communication

The importance of communication about the SNMRL cohort and the work being undertaken externally to a wider audience, was a consistent theme from both the survey and interview findings. The lack of a website or social media presence was felt to have been a missing element to communicating about the programme. Social media use is linked closely to nurses professional development, allowing nurses to connect with colleagues, develop knowledge and share information (Geraghty et al., 2021). A recent scoping review (Glasdam et al., 2022) demonstrated how nurses used social media during COVID‐19 as channels to gain and share information about COVID‐19, to highlight training and changes in delivery of care and redeployment, to profession‐promote and to educate people. With a lack of understanding about the value of nurse/midwife led research, contemporaneous sharing of information about the programme could have enhanced the visibility of nurse/ midwifery researchers and their role in research delivery. Future programmes or similar initiatives therefore need to consider raising the profile and visibility of the individuals, their programme of work and have an active presence on social media to encourage active dialogue and engagement of other health care professionals and the public.

### 6.5 Strengths and limitations of the study

A strength of the study was that the study evaluation surveys were anonymous and were sent out centrally through the Nursing and Midwifery Office. This encouraged the SNMRLs to be open about reporting challenges associated with the programme. The study employed a research fellow specifically to conduct the evaluation lead who was unknown to the SNMRL cohort. The consent rate of SNMRL invited to participate in interviews was high and the candour of participants indicates they felt psychologically safe to share their reflections. Limitations of the study evaluation included that evaluation of the hub meetings only commenced in year two of the programme, after the meetings moved on‐line due to COVID‐19. We were therefore unable to compare responses across the whole 3 years of the programme. In addition, due to the public health crisis some meetings were cancelled, therefore only five meetings were evaluated for attendee satisfaction. The programme survey was developed from feedback gained from SNMRLs in year one and although free‐text responses were available, the pre‐set questions may have limited respondents' options to voice additional challenges and benefits. The programme director was involved as part of the study team which may have influenced participants decision‐making about whether to participate, although the programme director was not directly involved in any aspects of recruitment or data collection. The timing of interviews in year two, meant that not all programme aims, and objectives had been fully achieved. The interviews were not therefore able to provide insight on the programme impact which will be reported on elsewhere.

### 6.6 Recommendations for future research

Further research needs to focus on the longer‐term impact of the programme on the SNMRL cohort and their continuation as senior research leaders. Support for the programme impact was reported by all stakeholder groups, however evaluation of subsequent programmes is required to ensure transferability of these results.

## 7 CONCLUSION

The 70@70 SNMRL programme was launched as both a developmental opportunity for the 70 SNMRLs, as well as being an initiative to support the future development of other nurses and midwives within the SNMRL's organisations. The study aimed to evaluate whether the NIHR SNMRL initiative achieved these programme development aims and found that the programme evaluated well from the perspectives of not only those on the programme, but also those overseeing their work within the NHS and the programme leads. Reported benefits included being part of a regional and national network, having protected time to address organisational needs and being able to access training and learning opportunities. The programme has international applicability as it demonstrates the value of an initiative to support the development of nurses and midwives to drive research capacity and capability building. Future programmes need to allow sufficient time for networking, facilitate collaboration amongst the cohort, provide clarity from the outset about programme aims and expectations and work with the NIHR to create accessible learning resources.

## AUTHOR CONTRIBUTIONS

The NIHR Nursing and Midwifery Office (NMO) developed the study concept and developed and piloted the surveys and facilitated distribution. CH designed the evaluation. RF conducted all stakeholder interviews and RF/CH/JCM all contributed to data interpretation. RF completed the initial drafts of the manuscript, with manuscript revisions from JM and CH. All authors reviewed and approved the final manuscript.

## FUNDING INFORMATION

This research received no specific grant from any funding agency.

## CONFLICT OF INTEREST STATEMENT

The authors declare that the research was conducted in the absence of any commercial or financial relationships that could be construed as a potential conflict of interest.

## Supporting information


Appendix S1–S3

## Data Availability

The data that support the findings of this study are available from the corresponding author upon reasonable request.
