# Supplementary material for: Assessing the success of a research leadership programme for senior nurses and midwives: A mixed methods programme evaluation
Source: Nurs Open. 2024 Jul 17;11(7):e2176. doi: 10.1002/nop2.2176 (PMC11255376; doi:10.1002/nop2.2176)
Supplement: Supplementary file 1 — Appendix S1–S3 [file NOP2-11-e2176-s001.docx]

**Appendix A: Year 1 Programme design survey**

1. If you were to give a score for your satisfaction with the programme overall, between 0-100, what would that be?

Explain why you have given this score (free text)

2. What works well for you? Please tick all that apply.

- Being part of a network
- Ringfenced time
- Control over own time and objectives
- Communication from NMO
- Resources on NIHR learn
- Other- free text

3. What doesn’t work so well? Please tick all that apply.

- Using NIHR Email address/using multiple email addresses
- Not enough opportunity to network as a national group
- The NIHR Learn forum
- Balancing the 70@70 role with other commitments
- Lack of direction/clarity from NIHR
- Not enough collaboration between regional hubs
- Organisational barriers within trusts
- Not enough transparency of what other participants are working on
- Varying levels of seniority amongst participants
- Confusion around modules - what they are and when added
- Central organisation of the programme
- Not enough support from other participants
- Not enough guidance on how to prepare for hub meetings
- Travel allowance
- Other- free text

4. Do you have a clear understanding of your role on the NIHR 70@70 programme? Please provide a score between 0-100.

Please explain your answer.

5. How have you found the NIHR Learn platform, i.e. access, ease of use, materials etc?

(Unsatisfactory – Excellent)

6. What topics would you like to see in the next two years?

**Appendix B: National meeting evaluation survey**

Please rank the following statements in relation to your experience of the session

(Strongly agree to strongly disagree)

1) The session was relevant and helpful to me as a research leader

2) The speakers were inspiring and provided me with useful insights

3) The content and topics covered were pitched at the right level

4) The session was thought provoking and made me reflect on my own role and

purpose as a research leader

5) I felt the session aligned well with the overall aims and scope of the 70@70

**Appendix C: Topic Guide for Programme Design Interviews**

**Openers:** Can you briefly describe your role in relation to the 70@70 Programme?

Can you briefly describe your background before starting on the 70@70 programme?

How many 70@70 members do you have in your organisation?

Were you involved in the programme set up? If so, can you describe your role in this?

Have you been you involved in the implementation and/or delivery of the programme? If so, how?

**Follow on questions:**

What would you say is the main purpose of the 70@70 Programme?

What do you think have been some of the challenges and barriers to the programme?

Which features of the programme do you like the most? (e.g. hubs, facilitators, NIHR Learn, national meetings, comms etc..). Which features do you not like?

What do you feel are the best things about the programme in terms of what it can offer?

What could make the programme better?

Can you provide any examples of successful collaborations that have come about as a result of the programme at an organisational, regional or national level? Have these been sustained?

Has the programme increased your awareness of other inspirational nurse leaders in research? If so, have you been able to share this awareness with colleagues locally/nationally?

How do you feel the 70@70 cohort has evolved collectively over the course of the programme? What are the reasons for this?

In terms of legacy or long term sustainability, are there any elements of the programme that you think will persist beyond the three years?

From an organisational perspective, how much has the Senior Nurse & Midwife Research Leaders programme raised the profile of NMs in terms of, for example relationships with a) R&D, b) Chief Nurses etc…?

Do you feel the culture surrounding research has changed within your organisation during your time on the programme?

Is there anything you would do differently in terms of set-up and delivery if you were involved in a similar programme in the future?

Overall, how well do you feel the programme has fulfilled its aim of helping senior NMs to increase research capacity and capability, support future research leaders and contribute to key NIHR priorities? Why?

Do you think the programme has demonstrated ‘value for money’? Examples?

Cohort member specific: Without the 70@70 programme, do you think you would have still achieved what you have done during your time on the programme?

Can you describe the impact that Covid-19 has had on the programme?

How well do you feel that the leadership team has responded to the requests of the cohort?

Is there anything else you would like to add?
